# Association between social capital and depression among older adults of different genders: Evidence from Hangzhou, China

**DOI:** 10.3389/fpubh.2022.863574

**Published:** 2022-08-12

**Authors:** Siyu Zhou, Kai Li, Atsushi Ogihara, Xiaohe Wang

**Affiliations:** ^1^School of Public Health, Hangzhou Normal University, Hangzhou, China; ^2^School of Medical Technology, Zhejiang Chinese Medical University, Hangzhou, China; ^3^Department of Health Sciences and Social Welfare, Faculty of Human Sciences, Waseda University, Tokorozawa, Japan

**Keywords:** social capital, depression, older adults, gender, trust, reciprocity, social network, social participation

## Abstract

In China, it is critical to help older adults cope with depression due to the emerging impacts of factors such as increased life expectancy and the “one-child” family planning policy. Meanwhile, differences in retirement age have different effects on health in older adults of different gender. The relationship of gender differences in social capital and depression across the elderly population was unclear. Focusing on this demographic, this study conducted a telephone survey to explore the relationship between social capital and depression. Referring to electronic medical records, we randomly selected 1,042 elderly respondents (426 men, 616 women) from four areas in Hangzhou. We used social capital measurements and the Geriatric Depression Scale (GDS-15) to assess social capital and depression, respectively, then employed a multivariate logistic regression and structural equation modeling to examine the associations between factors, along with a consideration of gender. This study was discovered that differences in both income and morbidity contributed to differences in social capital and depression. In our sample of elderly respondents, we also found gender-based differences in cognitive and structural social capital. Compared to men, women were more likely to attain higher social capital and less likely to develop depression. At the same time, social networking and social engagement had negative impacts on depression in women, which was not the case for men. We found that lower reciprocity (men and women), social work (men), and trust (women) indicated higher risks of depression. Reciprocity and social networks were significantly and negatively correlated with depression among male respondents; in the male model, factors of trust, reciprocity, and social participation had positive effects on reducing the risk of depression, while social networks had a negative effect. For elderly persons, these findings suggest that mental health is affected by differences in social capital caused by policy differences and cultural differences caused by gender differences.

## Introduction

The relatively affluent eastern part of China has an aging population. With the strict family planning policy (the one-child policy) and the advancement of urbanization, the proportion and number of older adults in China is set to increase rapidly, and this has resulted in tremendous changes in both health and social capital. Depression has become one of the most common disorders among older adults, and the phenomenon of social isolation has been recognized as a growing medical, social, and public health problem (1). Data from the 2013 survey of the China Health and Retirement Longitudinal Study (CHARLS) showed that 34% of community-dwelling older adults experienced depressive symptoms and the proportion of urban older adults with depressive tendencies was close to 25% (2). Depression is one of the most common health problems for older people worldwide, and depression in Chinese older adults appears to have some geographical variance and an era-defining characteristic. First introduced in 1980, China's “one-child” policy has since effectuated the “4-2-1” family composition (i.e., four grandparents, two parents, one child). By extension, an increasing number of Chinese older adults are living alone (3). This is a public health concern, as evidence shows that older persons with “empty-nests” are more likely to have depressive symptoms when compared to peers without this type of living arrangement (4). With socio-economic development and urbanization, the number of older adults that have moved from rural areas to cities has increased. Additionally, some studies have found that better economic status, more social support, and better health in China are the main reasons why the urban older people have a lower prevalence of depression than the rural older adults (5, 6). Apart from personal variables such as gender, age, occupation, marital status, and education level, factors such as behaviors, lifestyle, and self-care skills influence depression in the urban older people (7–9). Meanwhile, differences in retirement age have different effects on health in older adults of different gender. For example, women tend to have better health status than men as post-retirement age increases (10). The 5-year gap in retirement age is also an influential factor in the cumulative incidence of chronic disease, leading to higher health risks for older adults who retire at more advanced ages (11). In terms of social capital, different retirement ages are further associated with different changes in the social network between genders, though individuals who retire earlier are more likely to perceive community identities (12). By contrast, cognitive decline after retirement directly weakens social capital for elderly persons (13).

In recent years, the social capital of older people has become a global public health priority. Social capital means that “the efficiency of society can be improved by activating collaborative activities, such as trust, norms, and networks (14).” It has become clear that the weakening of social capital leads to an increased risk of depression in the older adults (15, 16). In China, scholars have recently begun to focus on gender differences in social capital and depression across the elderly population. Some important findings have been made in this direction. For example, one study found that retired female elderly persons in China increased their social capital by actively integrating into social organizations (17). In similar regard, both the neighborhood environment and cognitive social capital are particularly significant factors among the female elderly, but community social capital appears weaker in the urban male elderly when compared to female counterparts (18). Moreover, variations in social capital due to gender differences in the elderly are reflected in family relationships. Here, women are more likely to gain social capital from family members and relatives, while men are more likely to obtain social capital from friendships established in professional organizations (19). Given these factors, gender differences in depression are increasingly prominent among elderly persons in China. One study found that women who participated in social activities (especially religious activities) were more willing to reduce their risk of depression through social organizations (20). Due to changes in both the family structure and social capital, depression has also risen among elderly persons who desire more companionship from children, wherein female elderly persons who do not have the opportunity to engage in child care are at a higher risk of depression (21). In males, cognitive decline is a major cause of reduced social capital, and also contributes to an increased risk of depression (22).

Structural and cognitive social capitals are positively correlated with depression among the older adults in China (23). Past research indicates that cognitive social capital, which includes trust and reciprocity, is a major factor of depression (24). The social network hierarchy included in structural social capital was significantly associated with depression, whereas social participation was not associated with depression (25). A study based on the classification regression tree model found that social capital and depression had a combined relationship and implied that different dimensions of social capital would have combined effects on depression in older adults (26–28). Similarly, different environmental factors (e.g., upbringing or work culture) can affect social capital and increase symptoms of depression and anxiety (29). With such negative consequences, it is critical to address the impacts of social capital on mental health in the urban elderly. This is especially the case for the government, which should adopt policies that expand social capital for all elderly persons, as such provisions are known to both improve overall mental health and reduce the prevalence of mental health disorders (30). Evidence shows that mental health factors partially mediate the relationship between social capital and depression, and can also affect self-perception in the elderly (31). In areas with severe aging, suicide rates have been found to increase as a direct result of depression caused by insufficient social capital (32). Given these concerns, many countries and regions with large aging populations implement health intervention policies that aim to reduce mental illness. At the same time, social capital generates health differences under the influence of various factors. For the elderly, enhanced social capital promotes change in the external social environment and improves internal mental health, thereby enhancing social status, addressing gender differences, and targeting other factors that lead to variations in health status (33). Social capital can also be subdivided into the individual and community types; men tend to have advantages in both, which creates differences in self-rated health (34). It is also important to monitor the health impacts of online social capital in elderly populations, as the Internet has become a primary communication medium. While this form of capital improves health for some elderly persons, it may exacerbate existing inequities for those who do not interact online (35). In other words, social capital imposes additional health effects due to the introduction of new factors in the information age.

**Figure 1:**
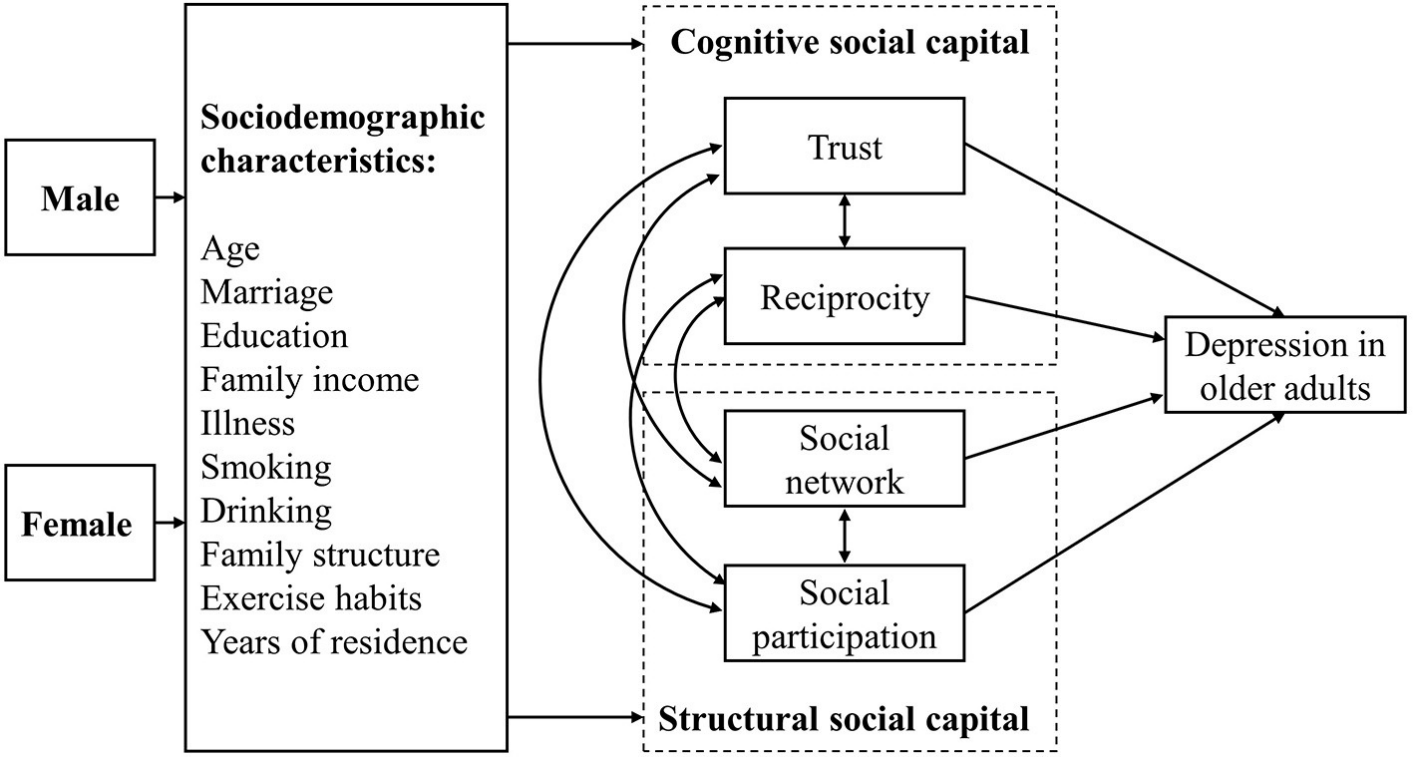
Hypothetical model.

The literature discusses many socioeconomic and social capital-related factors that affect depression in older adults, including explanations of the various interrelationships between those factors. A range of such influences pertain to gender. For example, gender-based social inequities in traditional society have enabled men to gain more social capital than women (36). However, elderly women are more willing to participate in community activities after retirement, which leads to better mental health vs. their male counterparts (37). The income gap between men and women also narrows after retirement; in China, women are most often the primary family financial managers at this time, and are also more willing to perform this duty, which further leads to differences in mental health between genders (38). Still, there is no gender-based explanation for the influence of social capital on depression, nor is there a suitable analysis of the logical relationship between these factors. Focusing on China, the 5-year gap in retirement age between men and women has created large differences in social capital during post-aging, especially when compared to the conditions in other countries. Based on this, we hypothesized that both cognitive social capital and structural social capital have different effects on depressive status in older adults depending on their sex. Figure 1 illustrates our hypothetical model.

## Methods

### Procedure and participants

A cross-sectional survey was conducted among older adults (Male aged ≥60 years, Female aged ≥55 years) in Hangzhou, Zhejiang Province, People's Republic of China. According to the 2015 Hangzhou Population Yearbook, 493,000 and 561,000 male and female retired elderly persons reside in Hangzhou, respectively. To achieve a confidence level of 99.5% margin of error and 50% response distribution, our survey required minimum sample sizes of 383 males and 383 females. Based on research experience, telephone surveys may incur 50% rates of loss to follow-up. In this study, we required at least 1,020 respondents, as we aimed for average sample sizes in four regions, including 300 in each region for a total of 1,200 respondents. Based on the male-to-female ratio across the elderly population in Hangzhou, we recruited 552 males and 648 females.

All surveys were conducted by family doctors and recorded by volunteer college students. Telephone survey training was conducted prior to survey initiation. The survey inclusion criteria were: (1) sign a contract with their family doctor; (2) men must be retired and over 60 years of age, while women must be retired and over 55 years of age; (3) can answer questions clearly. The survey exclusion criteria were: (1) refused to participate in the telephone survey; (2) unable to answer questions accurately. With the cooperation of primary medical service institutions (Tianshuiwulin Health Center, Changqing Chaoming Health Center, Zhalongkou Health Center, Kaixuan Health Center), we disseminated the questionnaire information to respondents *via* telephone. Thus, we received 1,042 responses (recovery rate of 86.8%), including 284 from Tianshui Wulin Health Center, 271 from Changqing Chaoming Health Center, 252 from Zhanongkou Health Center, and 235 from Kaixuan Health Center. The survey period was from October to December 2016.

### Demographic characteristics, health-related factors, and health status

Questions on gender, age, marital status, educational background, annual family income, illness, exercise regimens, smoking habits, drinking habits, family structure, and years of residence were included in the questionnaire (Table 1).

**Table 1:** Demographic characteristics of participants.

	**Category**	**Male (*n*)**	**%**	**Female (*n*)**	**%**
Age	Under 75	332	31.9	460	44.1
	75 or older	94	9.1	156	14.9
Marriage	Unmarried	4	0.4	6	0.6
	Married	370	35.5	504	48.4
	Divorced / widowed	52	4.9	106	10.2
Education	Primary school	108	10.4	218	20.9
	Middle school	218	20.9	332	31.9
	University	100	9.6	66	6.3
Family income	<90,000 yuan	112	10.7	206	19.8
	More than 90,000 yuan	314	30.1	410	39.3
Illness	With illness	138	13.2	162	15.5
	Without illness	288	27.6	454	43.6
Smoking	Smoking	136	13.1	34	3.3
	Not smoking	214	20.5	572	54.9
	Quit smoking	76	7.3	10	0.9
Drinking	Drinking	165	15.8	100	9.6
	Not drinking	175	16.8	454	43.6
	Quit drinking	86	8.3	62	5.9
Family structure	Have cohabitants	398	38.2	570	54.7
	live alone	28	2.7	46	4.4
Exercise regimen	Have	376	36.1	540	51.8
	Have not	50	4.8	76	7.3
Years of residence	<10 years	82	7.9	115	11.0
	10–30 years	256	24.6	382	36.7
	More than 30 years	88	8.4	119	11.4

*1. Family income: Based on the average annual family income in Hangzhou (88,421 yuan in 2016), the options were trimmed to “below 90,000 yuan” and “above 90,000 yuan.” 2. Illness: For persistent diseases, multiple answers were selected for hypertension, hyperlipidemia, diabetes, cancer, joint pain/backache/nerve pain, kidney disease, liver disease, obesity, gastrointestinal disease, eye disease, and other diseases under treatment. If participants were using any treatment they fell into the “with illness” category, and if not, they were in the “without illness” category*.

### Measures

#### Measures of depression

The Geriatric Depression Evaluation Scale (GDS-15) (Chinese version) was used in this study. It is one of the most used scales for depression screening in China (39). The GDS-15 is a simple test consisting of 15 questions. The subject answers “yes” (1 point) or “no” (0 points) to a situation related to depression. A total depression score is calculated as the sum of all scores for each item answered with “yes.” According to the GDS scoring standard, scores of 0–4 points indicate no depression, while scores of 5–9 points indicate mild depression, and scores of 10–15 points indicate severe depression.

#### Measures of social capital

Social capital was measured by simplifying a scale, which consisted of nine items of cognitive social capital and five items of structural social capital (Table 2) (40). Responses were provided on a five-point Likert scale, with “strongly agree,” “agree,” “neutral,” “disagree,” and “strongly disagree” corresponding to 5, 4, 3, 2, and 1 point respectively. Here, higher social capital scores indicate more social capital. This scale has been confirmed to be reliable and effective in surveys (Cronbach's α coefficient = 0.814) (41).

**Table 2:** Social capital scale.

	**Elements**	**Concept**	**Content**
Cognitive social capital	Trust	Strangers trust	Most people can be trusted
		Social atmosphere trust	The fallen item returns to its original owner
		Public staff trust	Public organization staff can be trusted
		Family doctor trust	Family doctors are reliable
		Neighbor trust	Neighbors can be trusted
	Reciprocity	Cared by neighbors	Neighbors are worried about others in addition to caring for themselves
		Mutual help atmosphere	Most residents help each other
		Caring about neighbors	Help him/her if the neighbor is in trouble
		Support for public welfare	You support what is beneficial to others
Structural social capital	Social network	Network diversity Network top Network differences	Total number of network member occupations Highest reputation among network members The highest reputation score of the network members minus the lowest reputation score
	Social participation	Number of organizations	The number of participants in social organizations
		Frequency of participation	Frequency of participation in events per year

The social network adopted the “Access to Social Capital” method (42). This method indirectly obtained the total number of social networks by asking the professional status of family members, friends, and acquaintances of the respondents based on their occupations. In traditional Chinese culture, different levels of professional prestige exist within the social network. Individuals who work in government, education, and healthcare have higher social recognition and professional prestige than those who work in business, services, and agriculture. Older persons believe that friends with high levels of social approval will bring more social resources to help them cope with difficulties during retirement. As such, friends with high social recognition can obtain higher social network scores. The main indicators include network diversity (total number of network member occupations), network top (highest reputation among network members), and network differences (the highest reputation score of the network members minus the lowest reputation score). Supplementary Table 1 shows the professional status scores of occupations in this study. The Professional Status List of Chinese Occupations shows 20 types of occupation. Network diversity refers to the total number of occupations a respondent reported (ranging from 0 to 20); the top range of the network was 0–95 points, and the network difference was 0–94 points. Factor analysis of the three variables led to a social network, and the scores of the three variables were standardized and added together to calculate the social network score. The Social Network Score Calculation Formula is as follows:


$$\begin{array}{ll} = & \overset{i}{\underset{i=n}{\sum }}\frac{\left(\alpha -\alpha_{\min}\right)\left(\alpha_{\max}-\alpha_{\min}\right)}{n}+\frac{\left(\beta -\beta_{\min}\right)\left(\beta_{\max}-\beta_{\min}\right)}{n}\\ + & \frac{\left(\gamma -\gamma_{\min}\right)\left(\gamma_{\max}-\;\gamma_{\min}\right)}{n}. \end{array}$$

where α = network diversity, β = top range of the network, and γ = network difference.

The number of participants in social organizations and participation frequency were calculated for social participation. Social organizations included political groups and associations; industry groups; volunteer circles; civic movements/consumer movements; groups related to religions, hobby, and sports, senior citizens' clubs; and production/employment. Respondents answered “yes (=1)” or “no (=0)” to whether they participated in an organization. The number of participations in activities for the older adults in each year is taken as the frequency of participation. This method is widely used in Asian population studies (43).

Social capital scores were categorized by quartiles: low, slightly low, slightly high, and high (44). These quartile scores are helpful for analyzing overall social capital levels and clarifying any differences in levels between genders (45, 46). The trust was divided into low (0–18 points, 28.6 per cent), slightly low (19–21 points, 25.1 per cent), slightly high (22–24 points, 31.9 per cent), and high (25 points, 14.4 per cent). Reciprocity was divided into low (0–12 points, 27.1 per cent), slightly low (13–16 points, 34.1 per cent), slightly high (17–19 points, 15.2 per cent), and high (20 points, 23.8 per cent). Social networks data were subjected to the normalization test (Shapiro-Wilk), and divided into low (0–0.31 points, 33 per cent), slightly low (0.32–0.64 points, 22.9 per cent), slightly high (0.65–0.93 points, 20.8 per cent), and high (0.94–3 points, 23.4 per cent). Social participation was divided into low (0–1 points, 33.2 per cent), slightly low (2–3 point, 48.6 per cent), slightly high (4–5 points, 15.2 per cent), and high (above 5 points, 5 per cent).

### Statistical analysis

We used *t*-tests and the one-way analysis of variance (ANOVA) to differentiate social capital scores for different social characteristics. We used the χ^2^ test to investigate associations between groups in terms of age, marital status, education, illness, smoking, drinking, family structure, exercise regimen, and years of residence. Moreover, we analyzed factors that influenced the impact of social capital on depression using the ordinary least squares (OLS) regression as a robustness analysis. Multivariate logistic regression models calculating odds ratios (ORs) and corresponding confidence intervals (CIs) were applied. Model 1 was not adjusted, while Model 2 was adjusted for age and educational background, and Model 3 included age, educational background, family annual income, exercise regimens, and years of residence. We tested for multicollinearity using the stepwise regression. After completing the collinearity diagnosis, the eigenvalue of the Model 1 regression model was 0.32, with a condition index of 2.29; the eigenvalue of the Model 2 regression model was 0.40, with a condition index of 2.53; the eigenvalue of the Model 3 regression model was 0.52, with a condition index of 3.87. We found no multicollinearity between variables. Finally, we performed structural equation modeling to analyze the effect of social capital on depression and distinguish differences in the path coefficients of social capital and depression between genders. IBM SPSS Statistics 20.0 and Amos 20.0 (IBM) were used as the analysis software.

## Results

### Descriptive results

**Figure 2:**
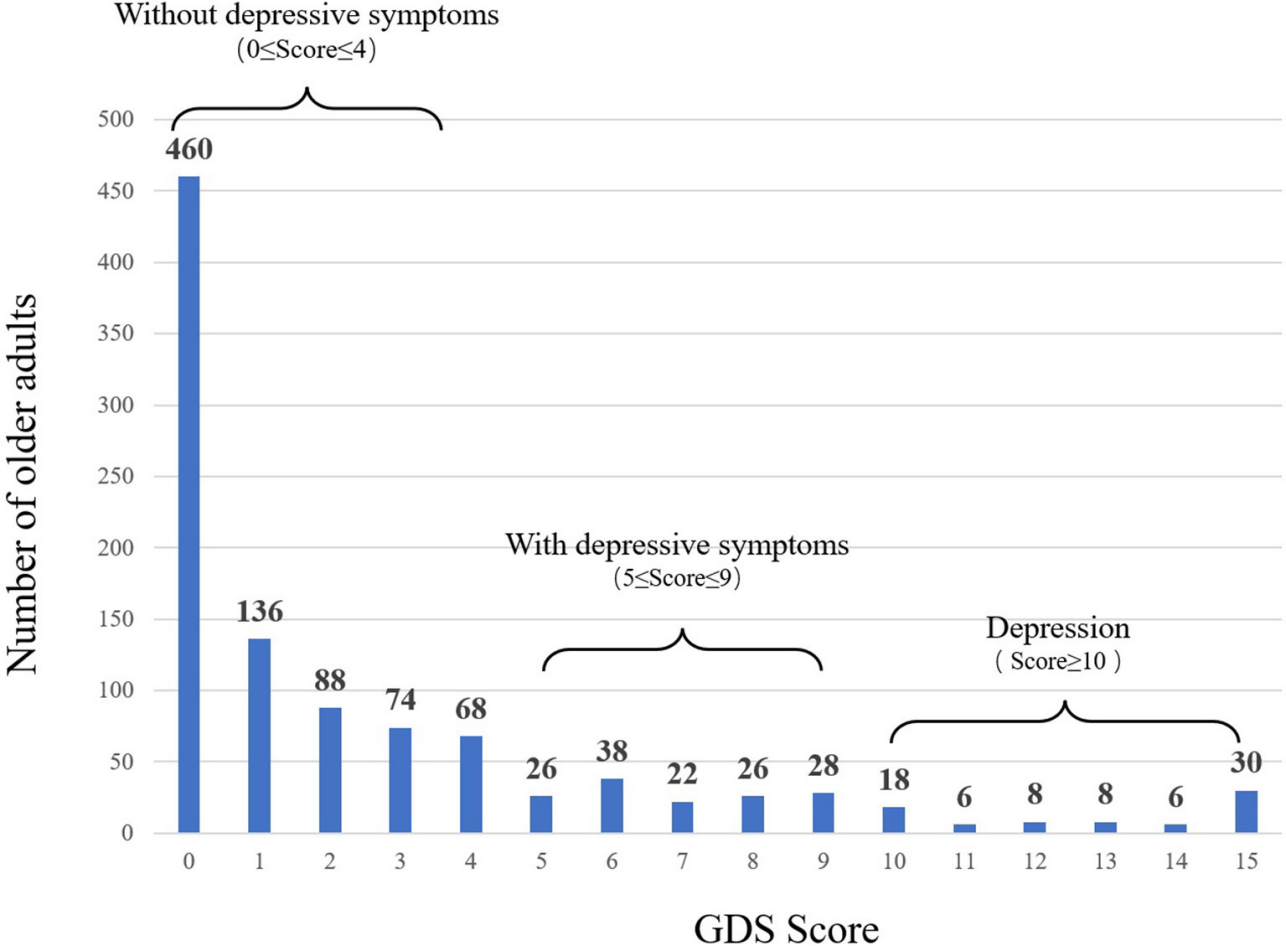
Depressive situation of older adults.

The average GDS score for the older adults was 2.65 ± 3.79 points. Based on the GDS scoring criteria, 0–4 was “without depressive symptoms,” 5–9 was “with depressive symptoms,” and 10–15 was “depression.” “With depressive symptoms” and “depression” were included in a depressive tendency group. The rate of depressive tendency was 20.7 per cent (Figure 2). Based on this GDS score, “without depressive symptoms” and “depressive tendency” were analyzed separately (47).

The “depressive tendency” group consisted of 37.4 per cent men and 62.6 per cent women. Based on the results of the χ^2^ test, a statistically significant association was found between the groups in terms of education, annual family income, illness, and years of residence. Compared to the “without depressive symptoms” group, those in the “depressive tendency” group had a relatively high proportion of participants who attended middle school and a relatively high proportion of family members with an annual income of <90,000 yuan, a relatively high incidence of illness, and who had lived locally for more than 10 years (Table 3).

**Table 3:** Comparison of depressive situations.

		**Without depressive symptoms**	**Depressive tendency**	**χ^2^**	***p***
	**Category**	***n* (%)**	***n* (%)**		
Gender	Male	334 (32.1)	92 (8.8)	0.33	0.566
	Female	492 (47.2)	124 (11.9)		
Age	Under 75	626 (60.1)	166 (15.9)	0.11	0.744
	75 or older	200 (19.2)	50 (4.8)		
Marriage	Unmarried	6 (0.6)	4 (0.4)	2.39	0.303
	Married	696 (66.8)	178 (17.1)		
	Divorced / widowed	124 (11.9)	34 (3.3)		
Education	Primary school	276 (26.5)	50 (4.8)	12.60	0.002
	Middle school	488 (46.8)	156 (15.0)		
	University	62 (6.1)	10 (0.9)		
Family income	<90,000 yuan	192(18.2)	126(12.1)	99.3	<0.001
	More than 90,000 yuan	634 (60.8)	90 (8.6)		
Illness	With illness	556 (53.3)	176 (16.9)	14.75	<0.001
	Without illness	260 (24.9)	40 (3.8)		
Smoking	Smoking	128 (12.3)	42 (4.0)	2.061	0.357
	Not smoking	628 (60.3)	158 (15.2)		
	Quit smoking	70 (6.7)	16 (1.5)		
Drinking	Drinking	224 (21.5)	42 (4.0)	5.376	0.068
	Not drinking	486 (46.6)	142 (13.6)		
	Quit drinking	116 (11.1)	32 (3.1)		
Family structure	Have cohabitants	770 (73.9)	198 (19.0)	0.626	0.429
	live alone	56 (5.4)	18 (1.7)		
Exercise regimen	Have	724 (69.5)	192 (18.4)	0.274	0.619
	Have not	102 (9.8)	24 (2.3)		
Years of residence	<10 years	171 (16.4)	26 (2.5)	8.638	0.013
	10–30 years	492 (47.2)	146 (14.0)		
	More than 30 years	163 (15.6)	44 (4.2)		

Social capital scores were affected by individual socioeconomic status, with differences in age, marital status, family income, and disease status (Table 4). The average score was 20.53 ± 3.63. The trust scores of people under the age of 75 were higher than those older than 75 years, the scores of married people were considerably higher than those of unmarried people, and the scores of those who were divorced or widowed were higher than those of unmarried people. The chronic illness scores of those with illness were lower than those without illness, and those participating in exercise regimens scored higher than those who did not. Reciprocity scores for people under 75 were lower than those of people aged 75 or older; scores for elementary school graduates were lower than those for university graduates or above; and people with an annual family income of <90,000 yuan scored lower than people receiving over 90,000 yuan.

**Table 4:** Relationship between basic attributes and social capital.

	**Category**	**Trust**			**Reciprocity**			**Social network**			**Social participation**		
		**Score**	**t**	***p***	**Score**	**t**	***p***	**Score**	**t**	***p***	**Score**	**t**	***p***
Gender	Male	20.89 ± 3.51	1.87	0.062	15.50 ± 3.61	−0.01	0.990	0.75 ± 0.59	−1.12	0.262	1.12 ± 0.99	0.64	0.522
	Female	20.28 ± 3.71			15.51 ± 3.50			0.81 ± 0.62			1.06 ± 1.09		
Age	Under 75	20.84 ± 3.54	3.51	<0.01	15.28 ± 3.58	−2.64	<0.01	0.95 ± 0.71	3.56	<0.01	1.13 ± 1.04	1.53	0.126
	75 or older	19.54 ± 3.77			16.23 ± 3.31			0.73 ± 0.57			0.96 ± 1.08		
Family income	Less than / 90,000	20.13 ± 3.66	−1.68	0.093	14.87 ± 3.53	−2.71	<0.01	0.86 ± 0.61	1.89	0.059	1.15 ± 1.06	0.92	0.356
	More than / 90,000	20.71 ± 3.61			15.78 ± 3.51			0.75 ± 0.61			1.06 ± 1.05		
Illness	With illness	19.85 ± 3.66	−7.02	<0.01	15.49 ± 3.51	−0.17	0.864	0.81 ± 0.67	1.26	0.206	1.09 ± 1.03	0.08	0.93
	Without illness	22.21 ± 2.96			15.55 ± 3.63			0.73 ± 0.42			1.08 ± 1.12		
Family structure	Have cohabitants	20.57 ± 3.66	0.87	0.384	14.45 ± 3.54	−1.31	0.189	0.78 ± 0.61	−0.83	0.407	1.11 ± 1.07	1.81	0.071
	Live alone	20.03 ± 3.25			16.24 ± 3.49			0.86 ± 0.59			0.78 ± 0.75		
Exercise regimen	Have	20.71 ± 3.63	3.06	<0.01	15.55 ± 3.50	0.71	0.476	1.01 ± 0.80	3.14	<0.01	1.16 ± 1.07	4.32	<0.01
	Have not	19.22 ± 3.42			15.21 ± 3.81			0.75 ± 0.57			0.56 ± 0.75		
	**Category**	**Trust**				**Reciprocity**				**Social network**				**Social participation**			
		**Score**	**SS**	**MS**	**F**	**Score**	**SS**	**MS**	**F**	**Score**	**SS**	**MS**	**F**	**Score**	**SS**	**MS**	**F**
Married			148.5	74.2	5.72 **		28.94	14.47	1.15 ns		0.01	0.01	0.02 ns		1.08	0.54	0.48 ns
	Unmarried	15.60 ± 2.30				13.60 ± 2.88				0.83 ± 0.47				1.20 ± 0.44			
	Married	20.67 ± 3.62				15.46 ± 3.55				0.78 ± 0.63				1.07 ± 1.05			
	Divorced / widowed	20.05 ± 3.57				15.86 ± 3.52				0.77 ± 0.54				1.19 ± 1.12			
Education			22.3	11.1	0.84 ns		100.47	50.23	4.05 *		0.13	0.06	0.17 ns		26.02	13.01	12.15**
	Primary school	20.68 ± 3.56				15.01 ± 3.54				0.79 ± 0.60				0.78 ± 0.84			
	Middle school	20.39 ± 3.66				15.62 ± 3.51				0.78 ± 0.62				1.19 ± 1.09			
	University	21.11 ± 3.71				16.75 ± 3.57				0.73 ± 0.59				1.53 ± 1.23			

^*^p <0.05,

^**^p <0.01, /, Currency RMB yuan; ns, no significance; SS, Sum of squared deviations; MS, Mean square.

Social network scores for people under the age of 75 were lower than those for people aged 75 or over, and those for people not participating in exercise regimens were lower than for those who participated in exercise regimens. Regarding the subjective economic situation, older adults who could afford living expense scored higher than those who could not. The average social participation score was 1.09 ± 1.15, and 137 people did not participate in regional organizations. The social participation scores of highly educated people were higher than those of the less educated. People not participating in exercise regimens scored lower than those who participated.

### The associations between socioeconomic factors, social capital, and depression for different gender groups

To further explore the impact of social capital on depression, we analyzed the impact by gender (Table 5). In China, there is a 5-year gap in retirement age between men and women (i.e., 60 for men and 55 for women). In the rural context, this gender-based difference leads to different changes in social capital for elderly persons, which then leads to changes in mental health (48). Model 1 was unadjusted; Model 2 controlled for age and educational background; and Model 3 controlled for age, educational background, family annual income, illness, family composition, exercise habits, and years of residence.

**Table 5:** Impact of social capital on depression.

		**Depression**					
		**Male**			**Female**		
		**Model 1** **OR (95%CI)**	**Model 2** **OR(95%CI)**	**Model 3** **OR(95%CI)**	**Model 1** **OR(95%CI)**	**Model 2** **OR(95%CI)**	**Model 3** **OR(95%CI)**
Trust	High	1.00	1.00	1.00	1.00	1.00	1.00
	Relatively high	1.35 (0.57–3.17)	1.38 (0.57–3.30)	1.02 (0.40–2.60)	1.53 (0.60–3.94)	1.69 (0.64–4.44)	1.26 (0.44–3.59)
	Relatively low	1.25 (0.54–2.90)	1.37 (0.58–3.22)	0.96 (0.38–2.43)	1.21 (0.45–3.25)	1.23 (0.45–3.37)	1.05 (0.37–2.98)
	Low	0.86 (0.38–1.92)	0.88 (0.38–2.01)	0.70 (0.29–1.67)	2.83 (1.13–7.07)*	3.07 (1.19–7.87)*	3.08 (1.14–8.29)*
Reciprocity	High	1.00	1.00	1.00	1.00	1.00	1.00
	Relatively high	1.35 (0.52–3.50)	1.35 (0.52–3.52)	1.29 (0.47–3.50)	3.37 (1.46–7.78)*	3.66 (1.55–8.61)*	3.70 (1.54–8.88)*
	Relatively low	1.78 (0.79–4.00)	1.83 (0.79–4.23)	2.58 (1.03–6.44)*	4.81 (2.16–10.67)*	5.06 (2.25–11.38)*	4.93 (2.15–11.29)*
	Low	2.71(1.25–5.87)*	2.57(1.16–5.70)*	2.97(1.30–6.81)*	2.26 (0.85–6.00)	2.75 (1.02–7.49)*	2.90 (1.03–8.12)*
Social network	High	1.00	1.00	1.00	1.00	1.00	1.00
	Relatively high	2.15 (0.82–5.62)	2.04 (0.76–5.44)	1.65 (0.59–4.59)	1.85 (0.93–3.69)	1.54 (0.76–3.14)	1.98 (0.92–4.21)
	Relatively low	2.31 (0.97–5.52)	2.06 (0.83–5.10)	1.61 (0.62–4.19)	2.18 (1.01–4.74)*	1.99 (0.90–4.41)	2.78 (1.18–6.56)*
	Low	2.85 (1.21–6.68)*	2.51 (1.05–6.09)*	2.84 (1.12–7.18)*	1.80 (0.85–3.80)	1.58 (0.73–3.41)	2.17 (0.94–4.99)
Social participation	High	1.00	1.00	1.00	1.00	1.00	1.00
	Relatively high	1.26 (0.45–3.53)	1.18 (0.40–3.50)	0.89 (0.27–2.92)	1.91 (0.85–4.30)	2.49 (1.05–5.89)*	3.24 (1.29–8.13)*
	Relatively low	1.26 (0.43–3.66)	1.15 (0.40–3.35)	0.74 (0.22–2.45)	1.94 (0.87–4.31)	2.68 (1.14–6.30)*	3.60 (1.43–9.04)*
	Low	1.80 (0.65–4.95)	1.82 (0.68–5.11)	1.31 (0.42–4.10)	1.73 (0.68–4.41)	1.91 (0.73–5.02)	2.28 (0.84–6.19)

OR, odds ratio; 95% CI, 95% confidence interval; ^*^p <0.05. Model 1: no adjustment; Model 2: age, educational background; Model 3: age, educational background, annual family income, illness, family structure, exercise habits, years of residence.

Model 1 shows that reciprocity and social networks were negatively correlated with depression in the male group. Compared with the high reciprocity and social network groups, low reciprocity and social network groups were associated with high levels of depression. Trust, reciprocity, and social participation were negatively correlated with depression in females. Compared with the high trust and social participation groups, the low trust and social participation groups were associated with high levels of depression.

Model 2 shows that reciprocity and social network remained significantly negatively correlated with depression, but the correlation degree was reduced in the male group. Trust, reciprocity, and social participation were significantly negatively correlated with depression, and the degree of correlation increased in the female group.

Model 3 shows that reciprocity and social network were significantly negatively correlated with depression in the male group. Trust, reciprocity, social network, and social participation were significantly negatively correlated with depression, and the degree of correlation increased in the female group.

### The relationships between social capital (i.e., trust, reciprocity, social network and social participation) and depression for different gender groups

**Figure 3:**
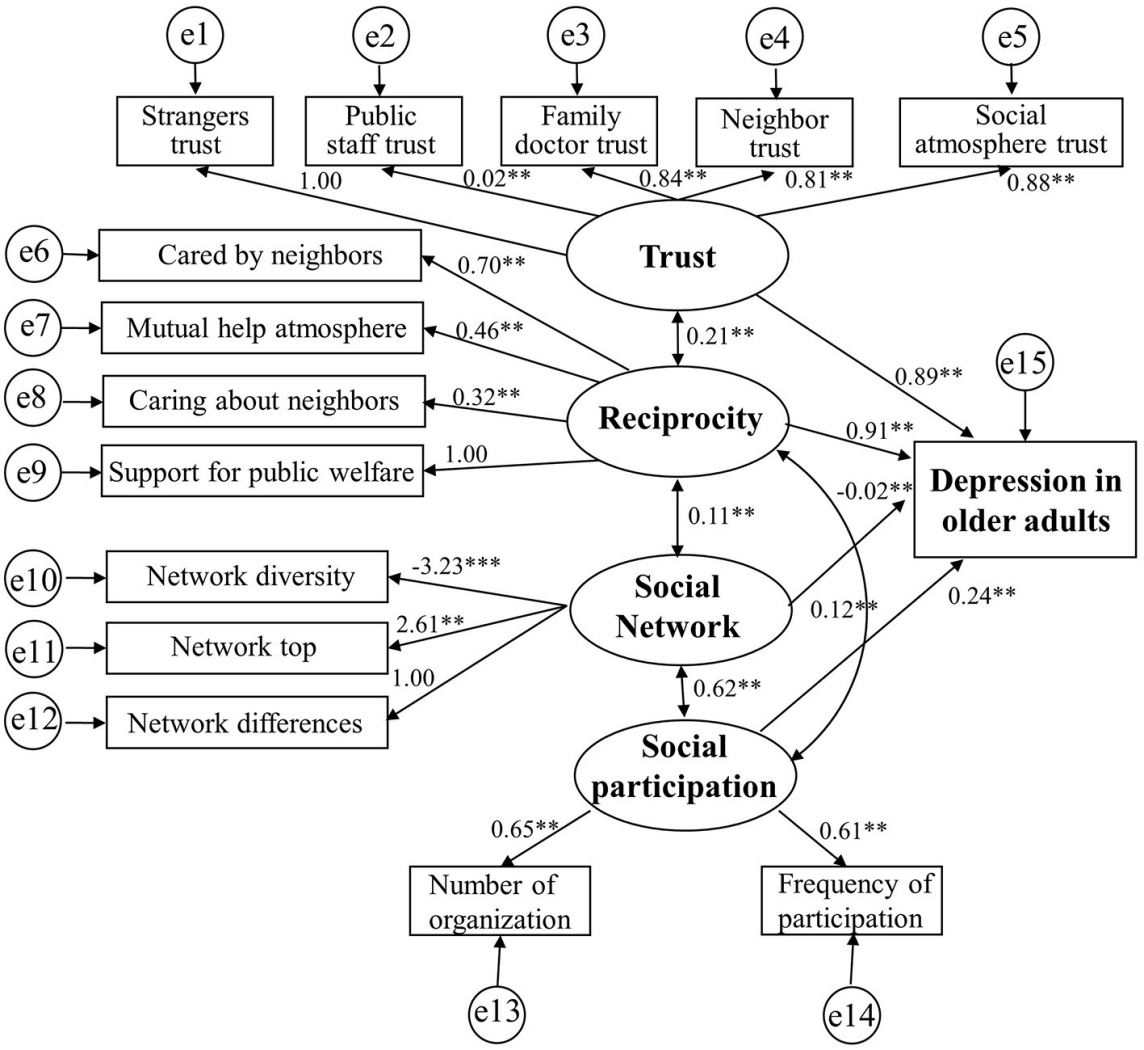
The relationship between social capital and depression in male model.

For the male model (Figure 3), the goodness-of-fit indices indicated a good fit in the analysis of the path model. The χ^2^/df value (322.643/83 = 3.88) was close to 3 and satisfied the model fit. The GFI (0.92) and CFI (0.91) values for the model were higher than 0.90, and the RMSEA value (0.073) was lower than 0.08. Trust, reciprocity, and social participation had a positive effect on reducing depression risk, but social networks seem to have a negative effect on depression. The effect of trust on depression was 0.89; the direct influence of reciprocity on depression was 0.91; the impact of social network on depression was −0.02, and the effect of social participation on depression was 0.24.

**Figure 4:**
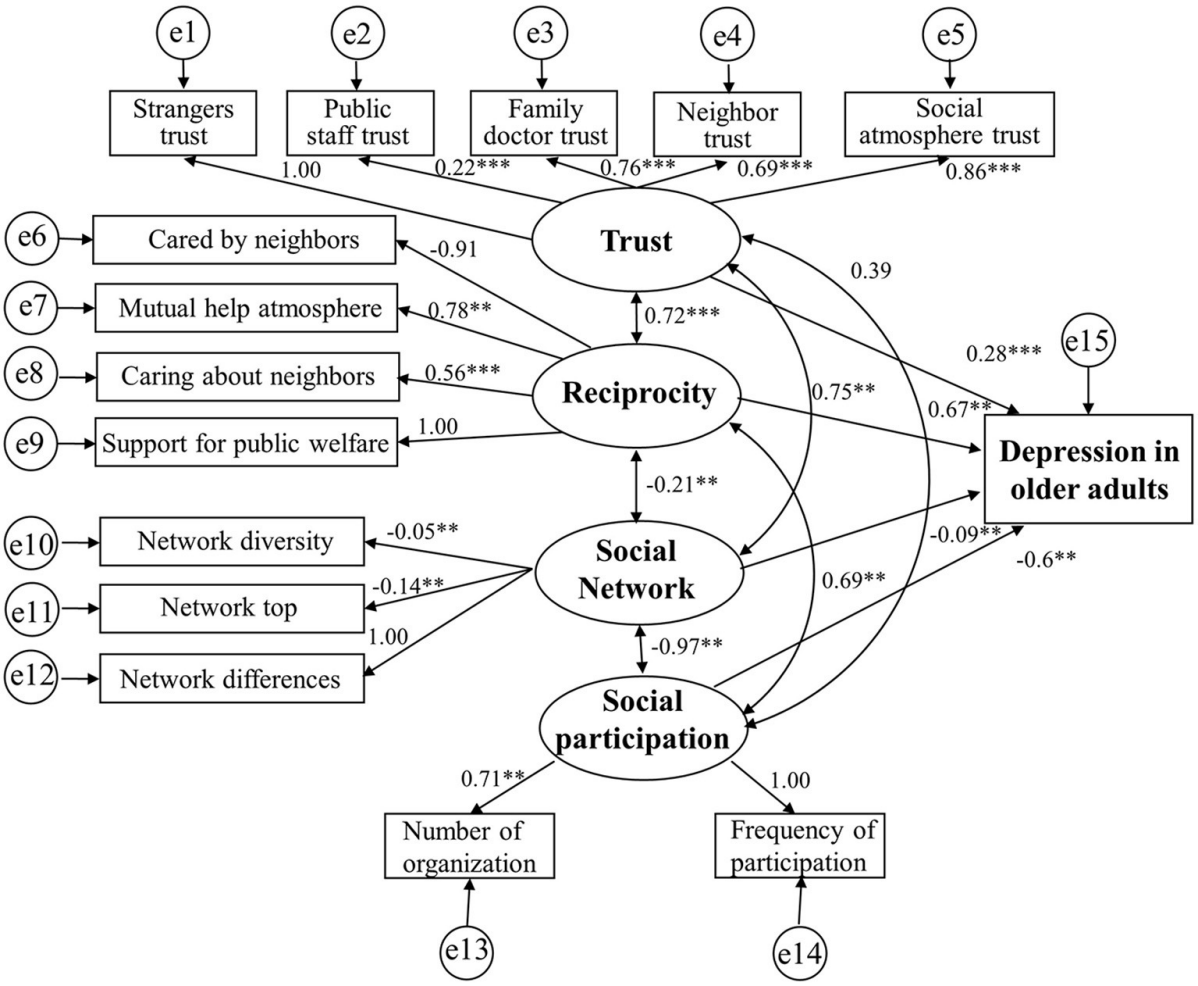
The relationship between social capital and depression in female model.

For the female model (Figure 4), the goodness-of-fit indices indicated a good fit in the analysis of the path model. The χ^2^/df value (394.380/81 = 4.87) ranged from 3 to 5, which satisfied the model fit, The GFI (0.89) and CFI (0.88) values for the model were higher than 0.85, and the RMSEA value (0.087) was close to 0.08. Trust and reciprocity had a positive effect on reducing depression risk. However, unlike those for males, social network and social participation had a negative effect on depression. The effect of trust on depression was 0.28; the effect of reciprocity on depression was 0.67; the effect of social network on depression was −0.09; and the effect of social participation on depression was −0.6.

## Discussion

The highlight of this study was the discovery that differences in both income and morbidity contributed to differences in social capital and depression. In our sample of elderly respondents, we also found gender-based differences in cognitive and structural social capital. Compared to men, women were more likely to attain higher social capital and less likely to develop depression. At the same time, social networking and social engagement had negative impacts on depression in women, which was not the case for men.

Previous studies have shown that social capital is closely related to health and well-being (49–51). In this context, older adults with lower social capital appear more likely to develop depression (52). With the rapid aging of the Chinese population, it is important to explore the risks of these types of illness, which are more likely to affect older adults. In this study, the sample produced a depression score of 6.24 ± 1.73, with 66.6% of respondents showing some form of depression based on GDS scores of 5 or higher. While urban populations are generally at a higher risk of depression, research has shown that younger high-income groups are at a lower risk than older high- and low-income groups (53). In this study, respondents with annual family incomes ≥ 90,000 yuan had a lower risk of depression than those with incomes <90,000 yuan. Given the increased Gini coefficient for income among elderly persons in China, there are also differences in their depression levels (54). Compared with income differences, social capital still appears to have greater impacts on depression in this demographic (55). Moreover, elderly persons in China are directly at a higher risk of depression due to the one-child policy, phenomenon of empty nesters, and weakening social network (56, 57). Although the elderly population in Hangzhou shows relatively high income levels, their risk of depression is still increasing due to rising prices and diminished income after retirement (58).

After adjusting our model for relevant variables, we found that social capital had different effects on depression between genders. This may be because social capital and depression are influenced by policies that pertain to gender. For example, men and women have different retirement ages, and thus enter the community at different times; therefore, the social network weakens more for men, which increases their risk of depression (59). On the other hand, elderly female empty-nesters significantly outnumber their male counterparts, and elderly women generally have lower incomes than elderly men, both of which decrease social participation and increase the risk of depression for women (60).

We also found significant differences in “trust” depending on age, marital status, chronic conditions, and exercise habits. This supports previous research showing that both “increased age” and the “prevalence of chronic diseases” are associated with decreased trust in older adults (61). In this context, sick elderly persons often prefer to stay at home in favor of going out for activities, as is particularly common in traditional Chinese culture (62). Research has also shown that married older adults generate higher “trust” indexes than single persons; in turn, older adults are more likely to engage in athletic activities when they have higher “trust” in public regional organizations (63). It is important to encourage this willingness, as elderly persons now have a greater range of choices due to both the increasing number of social organizations that provide relevant services and greater potential for interactions within various community organizations, all of which facilitate the attainment of higher social capital (64). We also found that women with low “trust” had a higher risk of depression in all adjusted models, which suggests that decreased “trust” is a risk factor for depression.

Next, we found a significant difference in “reciprocity” depending on age and annual family income. In relation to depression, our results showed that the risk increased in males as “reciprocity” decreased. In addition, the risk of depression in females was significantly lower only in the group with high “reciprocity” (adjusted Models 2 and 3). Given these findings, it is necessary to separately consider that the low “reciprocity” female group has a lower risk of depression than the slightly higher “reciprocity” male group. While increased social activity has expanded social networks for women, a recent rise in fraud has led to declined trust and reciprocity. In this regard, the main reason for declined structural social capital is the frequent occurrence of malignant social events (65). A survey on depression among older adults in rural China found that a stronger economy was associated with higher reciprocity (66). In urban areas, however, reciprocity has been influenced due to the fact that increasingly cold neighborhood relationships have replaced economic relationships (67). One researcher reported that “people with higher education build relationships through reciprocity,” and thus suggested that regional educational institutions should work to improve education levels in their respective areas (68). With the regular development of health education activities for the elderly in urban communities, mutual remuneration has gradually increased.

In addition, we found differences in the “social network” depending on age and exercise regimens. There was a significantly higher risk of depression for males in the low “social network” group, which suggests that a decrease in the social network is a risk factor for depression. This may be addressed through engagement in certain activities. For example, a previous study found that participation in walking clubs was an important social networking factor for older adults in Hong Kong (69). In similar findings, the diversification of sports clubs appears to broaden the social network for men more so than for women, wherein older men are more willing to make new friends through sports activities (70).

Further, we found a significant difference in “social participation” depending on the education background and presence/absence of exercise regimens. However, the only significant association was that the group with a slightly lower risk of depression had more females than the group with a slightly higher risk of depression (adjusted Models 2 and 3 with reference to “social participation”). This may be less associated with depression than “trust,” “reciprocity,” and “social networks.” “Social participation” refers to cases in which older adults participate in local community activities and organizations; it is closely related to social capital, and influenced by education background and family support (71). Since social organizations in traditional Chinese communities are primarily managed by government agencies, many elderly persons are reluctant to participate due to the lack of innovative activities (72). This limits the role of depression risk reduction through increased social participation. A previous study found that older persons who expanded their social networks also increased their frequency of organizational participation, and that social participation in older age was associated with community involvement from middle age (73). Likewise, more elderly persons are engaging in social participation as companies begin to offer diverse social activities in traditional communities (74).

As a new contribution to the literature, this study found evidence of a gender-based influence in the relationship between depression and social capital. Here, “trust and reciprocity” had a strong impact among elderly female respondents, which is not in accordance with international research. Multiple studies have reported that females are at a higher risk of depression than males (75, 76). As “trust” and “reciprocity” are strongly related to depression, it is thought that close relationships are significant factors for females. In the Chinese context, it is common for females to have weak friendships after retirement. In southern China, retired female elderly persons also play stronger family roles than their male counterparts, thus increasing their sense of family trust and willingness to participate in social activities (77). As for specific activities, one study reported that traditional Chinese square dancing was suitable for the female elderly, and could help reduce their risk of depression (78).

According to our structural equation model, the investigated social capital variables directly affected depression and influenced each other. There were also clear differences in the path coefficients between males and females. Due to gender-based differences in retirement policies and social security, men and women hold different community status, which creates variations in their social capital. Moreover, we found an association between “trust” and “reciprocity” in males with persistent critical illness (79). Males also had a greater overall risk of depression due to decreased “trust” and “reciprocity,” likely because Chinese males work longer than Chinese females and feel more uncomfortable upon retirement, which makes it difficult to quickly integrate into the community environment (80). By contrast, females adjust better to community life because they are more attuned to their families and retire earlier (81). It should also be mentioned that an increase in the social network did not reduce the risk of depression in the current study, as this finding directly contradicts existing reports (82, 83). This is likely because the social networks of Chinese retired older adults differ from those found in the workplace. Since older adults must therefore rebuild their community networks after retiring, efforts to increase one's social network in the employment context does not reduce the risk of depression.

In sum, our findings show that social capital has different impacts on depression in older adults depending on their gender. For elderly populations in China, the risk of depression will continue to rise due to both the increasing aging population and impact of delayed retirement policies. In fact, there is evidence that differences in retirement age will always lead to differences in social capital among older adults (84). The can be addressed through the Chinese government by gradually optimizing the structure of the labor force through delayed retirement policies and adjusting the influence of gender differences, both of which should help maintain health in the elderly (85).

This study also had limitations. First, all participants were 60 years of age or over and lived in Hangzhou, China, which limits generalizability. Although we found that social participation negatively impacted mental health in women, the specific elements of social participation require continued investigation. As such, future studies should both increase the number of participants and expand the scope of the survey area. Second, the activity ability of participants could not be measured by analyzing the relationship between their depressive situation and social capital. In future research, we plan to differentiate the effects of social capital on depression according to gender across the whole population. At the same time, we will consider how China's delayed retirement policy has impacted social capital and depression among the retired elderly.

## Conclusion

In this study, we investigated the relationship between depression and social capital among older adults in Hangzhou, China. Among the social capital indicators, “social network” was found to be significantly associated with depression in males, while “trust” and “reciprocity” were found to be significantly associated with depression in females. In addition, the results of this study suggest that relationships with familiar people, such as family members, neighbors, and local organizations, are particularly important for older adult women. To prevent depression in older adults in the community, it is necessary to improve their social capital through a social system that encourages social participation and efforts that focus on reciprocity. In terms of measures to enhance social capital, structural social capital (social network and social participation) is used as the main method, combined with the improvement of trust between neighbors, which ultimately reduces the risk of depression.

## Data availability statement

The raw data supporting the conclusions of this article will be made available by the authors, without undue reservation.

## Ethics statement

This study was approved by the Waseda University Ethics Committee (ID: 2018-278).

## Author contributions

XW contributed to the conception of the study. AO contributed significantly to analysis and manuscript preparation. SZ performed the data analyses and wrote the manuscript. KL helped perform the analysis with constructive discussions. All authors contributed to the article and approved the submitted version.

## Funding

This work was supported by the National Natural Science Foundation of China (No. 72104068), the Ministry of education of Humanities and Social Science project (No. 21YJC630180), and Philosophy and Social Science Foundation Project of Hangzhou (No. Z20JC100).

## Conflict of interest

The authors declare that the research was conducted in the absence of any commercial or financial relationships that could be construed as a potential conflict of interest.

## Publisher's note

All claims expressed in this article are solely those of the authors and do not necessarily represent those of their affiliated organizations, or those of the publisher, the editors and the reviewers. Any product that may be evaluated in this article, or claim that may be made by its manufacturer, is not guaranteed or endorsed by the publisher.
