# Supplementary material for: Association between social capital and depression among older adults of different genders: Evidence from Hangzhou, China
Source: Front Public Health. 2022 Aug 12;10:863574. doi: 10.3389/fpubh.2022.863574 (PMC9412187; doi:10.3389/fpubh.2022.863574)
Supplement: Supplementary file 1 [file Table_1.pdf]

1 Supplementary Tables

Supplementary Table 1

Table 1. Occupation and occupational prestige scores in the Position Generator method

Occupation	Score	Occupation	Score	Occupation	Score	Occupation	Score
Scientist	95	Government official	80	Administrative staff	53	Manufacturing worker	20
University professor	91	Primary/secondary school teacher	77	Policeman	52	Seller	15
Engineer	86	General manager	71	Nurse	48	Waiter	11
Lawyer	86	Business clerk	64	Driver	25	Part-timer	6
Doctor	86	Accountant	58	Cook	24	Farmer	1

Variables	Mean (SD)	Factor loading	Cronbach's *a*
Network diversity	4.41 (3.12)	0.885	0.872
Top range of the network	65.17 (28.75)	0.819	
Network difference	64.78 (29.01)	0.761	
Explained variance (%)	81.56		
